# Molecular Certification Laboratory of Arauco

**DOI:** 10.1186/1753-6561-5-S7-P181

**Published:** 2011-09-13

**Authors:** Paulina Núñez, Claudio Balocchi, Christian De Veer, Ximena Muñoz, Emilio Bustos, Philipp Bilabel, Mauricio Ramirez, Marcela Millar, Patricio Lavados, Jose Ordoñez, Liliana Villalobos

**Affiliations:** 1 Bioforest S.A. , Chile; 2 Forestal Celco, Chile; 3 Bosques Arauco S.A., Chile; 4 Forestal Valdivia S.A., Chile

## Objective

The main objective of the Laboratory is the Molecular Certification of the genetic material of Arauco, through the use of microsatellites (SSR).

## Methodology

For the selection of the markers, a reference population of 30 unrelated parents for each species of interest (Eucalyptus globulus and Pinus radiata) was used. In these populations, we tested 25 SSR for pinus and 38 SSR for eucalyptus and the best 12 markers were selected for each species. With the selected markers, a pattern or fingerprint of each operational clone was obtained, out of samples taken from the original material (tree or embryogenic mass). Against these patterns, a total of over 20,000 samples were compared for validation of various operational processes. The sampling is planned to get 90% confidence of detecting operational errors (contamination) greater than 10%. Since the laboratory should process a large number of samples, protocols have been developed to be simple and short. DNA is extracted with 96 commercial Qiagen kit, for different tissues (embryogenic cells, embryos, leaves, bark, etc.). Sometimes, extractions are doubled by mixing leaf tissue of P. radiata and E. globulus, which is possible because SSRs amplified in one species do not interfere with the other. The amount of sample used for the extraction process varies from 50 to 200mg, which yields to concentrations of 30 to 100ng/ul of DNA. Since the amount is enough for several analyses, there is no need to store the original samples. The amplification is performed in thermal cyclers AB 9700 with a single PCR program for each species, using 10 ng of DNA for P. radiata and 2-4ng DNA for Eucalyptus sp. The reading of the fragments is performed in AB 3130xl Genetic Analyzer, while the analysis and correction of the electropherograms is done with the GeneMapper 4.0 program. The fingerprint comparison is made with self developed software, which reduces the noise of size variation observed between samples from the same clone and the appearance of false alleles or silent alleles. All these noises are more noticeable in some clone-marker combinations.

## Results

Currently, Arauco has determined the pattern of 400 molecular clones of P. radiata and 200 clones of E. globulus. Thanks to the massive use of fingerprinting in the production and multiplication of clones, steps where mistakes are most likely have been identified and in many cases, errors have been repaired. During 2010, out of 4600 P. radiata samples corresponding to 170 clones, the rate of mistaken identity found was 5.6% in genetic field trials, 5.3% in the embryogenesis laboratory process and 8.4% in nurseries. In E. globulus for 3,000 samples analyzed, of 135 clones, the mistaken identity rate was found to be 7.4% in genetic field tests, 8.3% in clonal orchards and 7.1% in nursery samples.

